# BnTIR: an online transcriptome platform for exploring RNA‐seq libraries for oil crop *Brassica napus*

**DOI:** 10.1111/pbi.13665

**Published:** 2021-07-28

**Authors:** Dongxu Liu, Liangqian Yu, Lulu Wei, Pugang Yu, Jing Wang, Hu Zhao, Yuting Zhang, Shuntai Zhang, Zhiquan Yang, Guanqun Chen, Xuan Yao, Yanjun Yang, Yongming Zhou, Xuemin Wang, Shaoping Lu, Cheng Dai, Qing‐Yong Yang, Liang Guo

**Affiliations:** ^1^ National Key Laboratory of Crop Genetic Improvement Huazhong Agricultural University Wuhan China; ^2^ Hubei Key Laboratory of Agricultural Bioinformatics College of Informatics Huazhong Agricultural University Wuhan China; ^3^ Hubei Hongshan Laboratory Wuhan China; ^4^ College of Humanities & Social Science Huazhong Agricultural University Wuhan China; ^5^ Department of Agricultural, Food and Nutritional Science University of Alberta Edmonton AB Canada; ^6^ Department of Biology University of Missouri‐St. Louis St. Louis MO USA; ^7^ Donald Danforth Plant Science Center St. Louis MO USA

**Keywords:** *Brassica napus*, transcriptome, Co‐expression, database

With the increasing availability of massive transcriptome data in plants, it is possible to construct a comprehensive database with multiple transcriptome data and online imputation tools. Online databases with integrated, multifaceted functions for exploring published RNA‐seq libraries or microarrays are available for several plant species (Darwish *et al*., 2013; Xia *et al*., 2017; Zhang *et al*., 2020), helping researchers take advantage of the vast collection of public data sets. *Brassica napus* (AACC, 2n = 38) is one of the most important oil crops, originating from a spontaneous hybridization between *Brassica rapa* (AA, 2n = 20) and *Brassica oleracea* (CC, 2n = 18) (Chalhoub *et al*., 2014). Recently, an online transcriptome database has been built in *B. napus* (Chao *et al*., 2020). This database includes transcriptomes from different laboratories with various genotypes and growth conditions, which affects the usability. A database with comprehensive transcriptomes from one *B. napus* genotype is greatly needed.

To investigate the dynamic gene expression during *B. napus* development and provide an easy access visualization of the expression levels of *B. napus* genes, we performed RNA‐seq for 91 different tissue samples including root, seedling, stem, 1^st^‐23^rd^ leaves from the main branch, flower buds (2 and 4 mm), flower tissues (sepal, petal, filament, and pollen), silique walls (2–60 day after flowering (DAF), with 2‐day intervals) and seeds (14–64 DAF, with 2‐day intervals) (Figure 1a). Three biological replicates were set up for all tissues. Totally, 6.27 billion high‐quality reads were generated using the Illumina sequencing platform and then mapped to the ZS11 reference genome (Song *et al*., 2020). On average, 96.43% of the reads were mapped, which were further used to calculate the normalized gene expression level as transcripts per million mapped reads (TPM). The sequencing data of the biological replicates were of high quality, with Pearson correlation coefficients (*R* ^2^) > 0.90. Then, a *B. napus* transcriptome database BnTIR (http://yanglab.hzau.edu.cn/BnTIR) was built by using a comprehensive high temporal resolution of developmental transcriptomes of 91 different tissues (Figure 1b).

**Figure 1:**
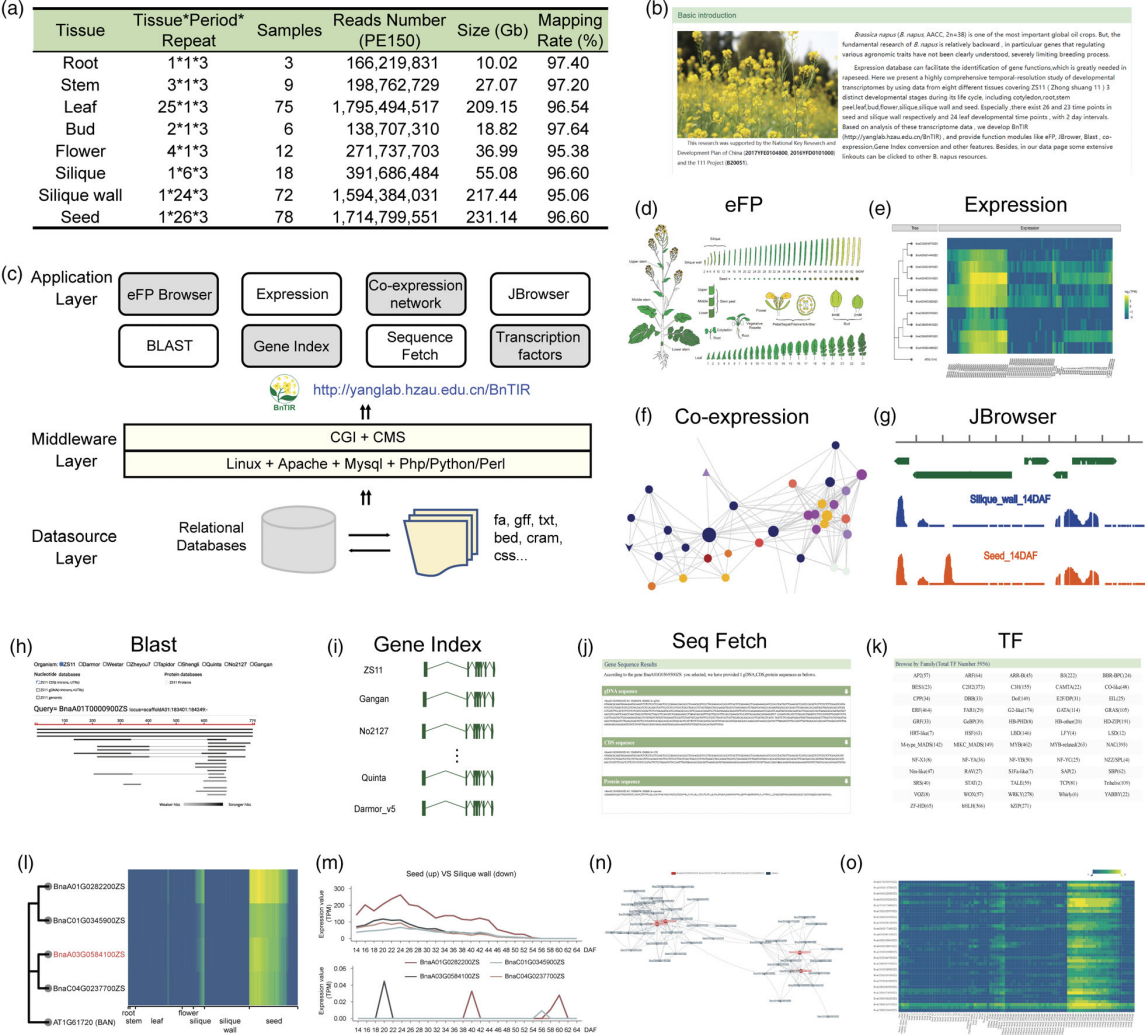
BnTIR is a refined transcriptome platform for *Brassica napus*. (a) Summary of statistics for transcriptome sequencing of diverse tissue types of *B*. *napus*. (b) The homepage of BnTIR. (c) Architecture of BnTIR database, including data source layer, middleware layer, and application layer. (d) eFP viewer. (e) Tissue expression viewer. (f) Co‐expression viewer. (g) JBrower viewer. (h) Blast module. (i) Gene index module. (j) Sequence fetch module. (k) Transcription factor module. (l–o) A case of application of BnTIR, including the phylogenetic tree and expression profile of *BAN* and their four *B. napus* homolog genes (l); expression comparison of *B. napus BAN* homolog genes in seed and silique wall (m); the co‐expression network of homolog genes of *BAN* (n); and expression profile of co‐expressed genes of *BAN* (o).

BnTIR was built on Apache web server (http://www.apache.org). All genomic data, gene expression, homologues, co‐expression network and TFs were organized and stored in MySQL database (https://www.mysql.com) (Figure 1c). The BnTIR could provide retrieval capabilities for the relevant expression data by the *B. napus* or *Arabidopsis* genes. The homepage allowed users to access many common tasks including eFP (electronic fluorescent pictograph) (Winter *et al*., 2007), expression, co‐expression, JBrowser, tools, download and help (Figure 1b). The external link modules contained TAIR (https://www.arabidopsis.org/index.jsp), BnPIR (http://cbi.hzau.edu.cn/bnapus) and BRAD (http://brassicadb.org/brad/index.php), attaching to the homepage for users to get more information.

The eFP viewer displays the selected gene expression pattern by dynamically colouring the tissues of a pictographic representation of *B. napus* plants according to gene expression levels based on TPMs (Figure 1d). On the gene expression search page, the gene expression information could be fetched by entering the gene ID, gene region or gene index (Figure 1e). The results include basic description of the genes and their expression profiles. After choosing individual gene or tissue of interest, the results could be displayed by three output forms including heatmap, line chart and boxplot. The gene expression results also include the expression profiles of the homologue genes. All data could be right‐clicked on selected genes and then clicked on the ‘Get Data’ button to see how the selected genes are expressed. In co‐expression module, the gene co‐expression network could be generated by entering the interested gene ID in the search area, by setting several parameters such as search depth and threshold (Figure 1f). The transcript view gives a graphical overview of features associated with a contig in the integrated JBrowser viewer (Figure 1g), which is a genome browser with a fully dynamic AJAX interface. The Blast module allows users to compare the query nucleotide or protein sequences with *B. napus* (including 11 genotypes), *B. rapa*, *B. oleracea* and *B. nigra* reference genome (Figure 1h). In Gene index interface, users could click on the gene ID to obtain the orthologs in other ten *B. napus* genomes. (Figure 1i). The reference sequences of genes are obtained directly through the seq fetch tool, including genome sequence, CDS sequence and protein sequence, which could be directly viewed and copied by clicking ‘fa’ button (Figure 1j). According to PlantTFDB, the information of 58 TF families in *B. napus* is also provided in the tool interface (Figure 1k; http://planttfdb.gao‐lab.org/). Then, a transcriptional regulation network was constructed, and ‘TF regulation network’ module was added in ‘Tools’. The downstream target genes or upstream transcription regulators could be retrieved according to the input genes. Furthermore, an expression heatmap could be created by entering a list of gene IDs in a very customizable way. On the download page, users could enter a gene ID or chromosome region of *B. napus* and all the relevant genes with their tissue expression levels would be shown at the same time on the results output page. The materials and methods, sample information, specifications, pipeline and contact are displayed in help interface.

Here, we present a case that demonstrates the application of BnTIR. *BAN* (*BANYULS*, AT1G61720) encodes key enzyme in procyanidin (PA) biosynthesis in *Arabidopsis* seed coat (Devic *et al*., 1999; Xie *et al*., 2003). After entering the *Arabidopsis* gene ID, the phylogenetic tree and expression profile of *BAN* and their four *B. napus* homolog genes were generated in expression module, which were highly expressed at the beginning of the seed development and followed by decreasing expression (Figure 1l). The comparison of line chart showed that *B. napus BAN* homologs were expressed in seed but silenced in silique wall (Figure 1m). Forty‐three genes were co‐expressed with *BnaBAN* (Figure 1n), and three of them encoded leucoanthocyanidin dioxygenase, which were involved in proanthocyanin biosynthesis. These results indicated that the co‐expressed genes may play a potential role in flavonoid biosynthesis. After that, an expression heatmap of these forty‐three co‐expression genes was generated in heatmap module, and those genes showed a consistent expression tendency with *BnaBAN* (Figure 1o). The homologue genes of *BnaBAN* in other genomes could also been fetched in gene index module, and those gene sequences (genomic, CDS and protein) were retrieved in seq fetch tool. Further, the alignment information among those gene sequences could be checked in BLAST module.

In summary, BnTIR provides a useful platform with user‐friendly, integrated, multifaceted functions for exploring the *B. napus* RNA‐seq libraries. These resources will help advance our understanding of the complex architecture of the regulatory mechanisms that govern biological processes in the undifferentiated polyploid genome of *B. napus*. Furthermore, BnTIR will be developed with more applications to provide better service for the research community. There are several future applications by using this comprehensive transcriptome data set including (i) improving the gene annotation of ZS11 by predicting the new genes, (ii) predicting the candidate genes in QTL or GWAS region, (iii) isolating new house‐keeping genes, (iv) discovering tissue‐specific genes and promoters and (v) analysing expression differentiation of homologs. In future, a greater variety of new RNA‐seq data such as transcriptome under various stress conditions will be available in BnTIR. Also, new functions and analysis tools will be added to BnTIR for the convenience of users such as (i) Experimental‐Search using keywords and (ii) Neighborhood‐Gene‐Expression‐Search to query information.

## Conflicts of interest

The authors declare no conflicts of interest.

## Authors contributions

L.G., Q.‐Y.Y., C.D. and S.L. designed the research. L.Y., P.Y. and S.L. performed the experiments and collected the samples. D.L., L.Y., L.W., J.W., H.Z., Y.Z. and Z.Y. performed the bioinformatics analysis and developed the BnTIR. Transcriptome data were deposited in NCBI under BioProject ID: PRJNA722877. S.Z. and Y.Y. designed the cartoon images of *B. napus* tissues. D.L., L.Y., and C.D. wrote the manuscript. L.G., Q.‐Y.Y., C.D., S.L., G.C. and X.Y. discussed the study and revised the manuscript. All authors read and approved the manuscript.
